# Mitogen activated protein kinase phosphatase-1 prevents the development of tactile sensitivity in a rodent model of neuropathic pain

**DOI:** 10.1186/1744-8069-8-34

**Published:** 2012-04-27

**Authors:** Christian Ndong, Russell P Landry, Joyce A DeLeo, Edgar Alfonso Romero-Sandoval

**Affiliations:** 1 Dartmouth Medical School, Department of Anesthesiology, Lebanon, N H, USA; 2 Department of Pharmacology/Toxicology, Dartmouth Medical School, Hanover, N H, USA; 3 (current affiliation) Department of Biology, Emmanuel College, Boston, MA, USA; 4 (current affiliation) Department of Bioengineering, Thayer School of Engineering at Dartmouth, Hanover, N H, USA; 5 One Medical Center Drive, DHMC – HB7125, Lebanon, NH, 03756, USA

**Keywords:** Phosphatases, MKP-1, Spinal cord, p38, Kinases, Allodynia, Nanoparticle, Nanotechnology

## Abstract

**Background:**

Neuropathic pain due to nerve injury is one of the most difficult types of pain to treat. Following peripheral nerve injury, neuronal and glial plastic changes contribute to central sensitization and perpetuation of mechanical hypersensitivity in rodents. The mitogen activated protein kinase (MAPK) family is pivotal in this spinal cord plasticity. MAPK phosphatases (MKPs) limit inflammatory processes by dephosphorylating MAPKs. For example, MKP-1 preferentially dephosphorylates p-p38. Since spinal p-p38 is pivotal for the development of chronic hypersensitivity in rodent models of pain, and p-p38 inhibitors have shown clinical potential in acute and chronic pain patients, we hypothesize that induction of spinal MKP-1 will prevent the development of peripheral nerve-injury-induced hypersensitivity and p-p38 overexpression.

**Results:**

We cloned rat spinal cord MKP-1 and optimize MKP-1 cDNA *in vitro* using transfections to BV-2 cells. We observed that in vitro overexpression of MKP-1 blocked lipopolysaccharide-induced phosphorylation of p38 (and other MAPKs) as well as release of pro-algesic effectors (i.e., cytokines, chemokines, nitric oxide). Using this cDNA MKP-1 and a non-viral, *in vivo* nanoparticle transfection approach, we found that spinal cord overexpression of MKP-1 prevented development of peripheral nerve-injury-induced tactile hypersensitivity and reduced pro-inflammatory cytokines and chemokines and the phosphorylated form of p38.

**Conclusions:**

Our results indicate that MKP-1, the natural regulator of p-p38, mediates resolution of the spinal cord pro-inflammatory milieu induced by peripheral nerve injury, resulting in prevention of chronic mechanical hypersensitivity. We propose that MKP-1 is a potential therapeutic target for pain treatment or prevention.

## Background

Chronic pain affects 116 million Americans and is estimated to cost over $560–635 billion per year (including health care and lost productivity) in the U.S. [1]. A large population of chronic neuropathic pain patients does not experience adequate relief with current treatments [2,3]. Therefore, identification of novel targets that allow the development of novel, more efficacious drugs is necessary.

Following peripheral insults such as nerve injury, several changes take place in the spinal cord, including glial responses and neuronal (central) sensitization. Both phenomena are associated with chronic hypersensitivity. Among the major pathways that determine the pro-algesic/pro-inflammatory phenotype in spinal cord following peripheral nerve injury are the MAPK pathways. Phosphorylation of MAPKs and the subsequent production of pro-inflammatory effectors in spinal cord are instrumental in the genesis and maintenance of acute and chronic pain [4]. The mechanisms by which peripheral MAPK phosphorylation results in allodynia (nociceptive response to innocuous stimuli) involve, at least in part, the production of pro-algesic effectors that sensitize spinal cord neurons and reinforce glial pro-inflammatory phenotypes [5,6]. Additionally, MAPKs expressed in neurons lead to increased neuronal excitability, contributing to the acute phase and long-lasting central sensitization in pain conditions [5]. Therefore, peripheral nerve-injury-induced MAPK dysregulation is integral for the perpetuation of pain [4].

Among MAPKs, p38 has particular clinical relevance. Drugs with novel mechanisms of action, such as inhibition of p-p38 (phosphorylated p38), have shown potential to treat postoperative pain [7] and neuropathic pain [8] in humans. Similarly, the deactivation of spinal p-p38 reverses peripheral nerve-injury-induced hypersensitivity [9,10] and persistent postoperative pain in rats [11].

In the periphery, p38 phosphorylation is instrumental in the development of inflammatory processes involving the efficacious production of pro-inflammatory cytokines [12], a mechanism also observed in spinal cord in pain models [13]. These peripheral processes are limited under normal conditions by natural regulators such as MAPK phosphatase-1 (MKP-1), leading to resolution of inflammation [12], a phenomenon that seems disrupted in spinal cord in chronic pain conditions where MAPKs are persistently phosphorylated.

Our laboratory has identified MKPs as potential analgesic targets that may control spinal MAPK phosphorylation [14]. MKPs are the major regulators of MAPKs and possess substrate specificity; thus, for example, MKP-1 preferentially regulates p-38 and, to a lesser extent, c-Jun N-terminal kinase (JNK) [10]. Thus far, the role of MKPs has not been elucidated in pain models. We hypothesized that spinal induction of MKP-1 will prevent peripheral nerve-injury-induced mechanical allodynia in a rat model. First, and due to the low efficacy for in vitro transfections using primary cells, we optimized our genetic tools using an immortalized cell line. We preferred to use a microglial cell line, BV-2 instead of a non-central nervous system cell line since our hypothesis is tested in vivo in the spinal cord. Second, once our spinal cord MKP-1 cDNA was optimized in vitro, we used a nanoparticle approach and *in vivo* spinal cord transfections to induce MKP-1 and tested its effects on downstream pro-algesic molecules.

## Results

### Spinal MKP-1 expression in naïve rats, and in rats undergone sham or L5 nerve transection surgery

**Figure 1:**
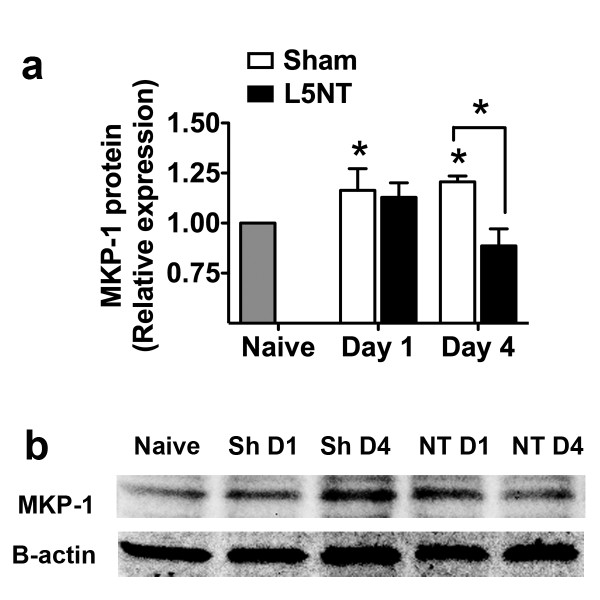
**MKP-1 expression in spinal cord following peripheral nerve injury.** Quantification of MKP-1 protein (**a**) and representative Western blot of MKP-1 expression (**b**) in L5–L6 spinal cord of naïve rats and of rats that have undergone sham (Sh) or L5 nerve transection (L5NT or NT) surgery (*n* = 3–6) on postoperative days 1 (D1) and 4 (D4) . *P < 0.05 for sham versus naïve on postoperative days 1 and 4, also between sham and L5 nerve transection groups on postoperative day 4. All data are expressed as mean ± s.e.m.

We sought to determine the expression level of MKP-1 in rats that underwent L5 nerve transection or L5 nerve-exposure sham surgery. Compared to the naïve group, the sham group displayed an increase of spinal MKP-1 protein at postoperative days 1 and 4. In contrast, spinal MKP-1 following L5 nerve transection did not differ from the naïve group on postoperative days 1 or 4, and was significantly lower than spinal MKP-1 in the sham group on postoperative day 4 (Figures 1a, 1b). We hypothesize that peripheral nerve injury prevents an increase in MKP-1, which allows p38 to be sustainably phosphorylated [13]. Therefore, we tested whether restoration of spinal MKP-1 in neuropathic pain conditions limits p-p38 and the downstream production of pro-inflammatory products and reduces tactile sensitivity.

### In vitro overexpression of MKP-1 in microglial cells

We cloned the rat MKP-1 cDNA from rat spinal cord homologous to the cloned MKP-1 (NM_053769) and first tested its functional effects inducing stable overexpression in an immortalized mouse BV-2 microglial cell line, as previous optimization to its use in in vivo experiments. There were two reasons to use BV-2 microglial cells for this optimization process. First, the transfection of primary cells is challenging, results in cell death and its efficiency is poor (30-40%) with virtually no advances in new technologies in the last decade [15,16]. Second, we consider more relevant the use of a cell line that is derived from central nervous system cells, microglia, instead of from peripheral origins (HELA, CHO, etc.).

**Figure 2:**
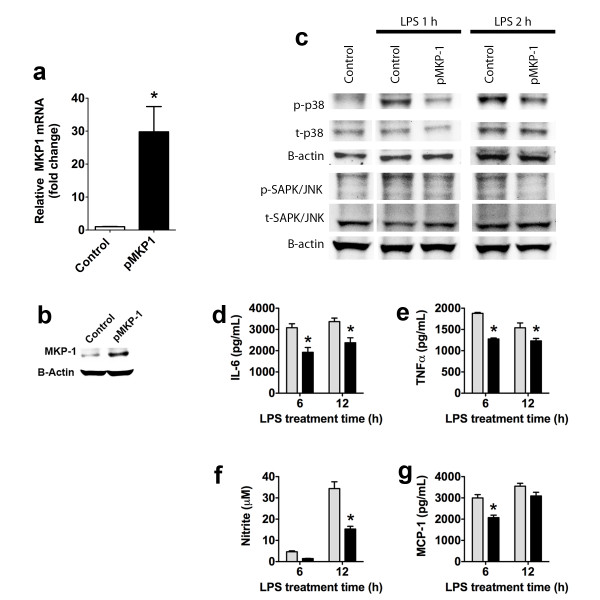
***In vitro*characterization of rat MKP-1 overexpression.** MKP-1 mRNA levels (**a**, qRT-PCR) and Western blot of MKP-1 protein (**b**) in normal BV-2 cells and stably MKP-1 overexpressing BV-2 cells. (**c**) Representative Western blots of p-p38, and p-JNK expression in BV-2 cells stably overexpressing MKP-1 following 1 or 2 h incubation in medium (control alone) or lipopolysaccharide (LPS) stimulation (1 μg/ml). IL-6 (**d**), TNF-α (**e**), nitrite (**f**), and MCP-1) (**g**) release in control (normal) and stably expressing MKP-1 BV-2 cells challenged with LPS (1 μg/ml) for 6 or 12 h. Results are expressed as mean ± s.e.m. of three experiments in triplicate. *P < 0.05 vs. control BV-2 cells.

**Figure 3:**
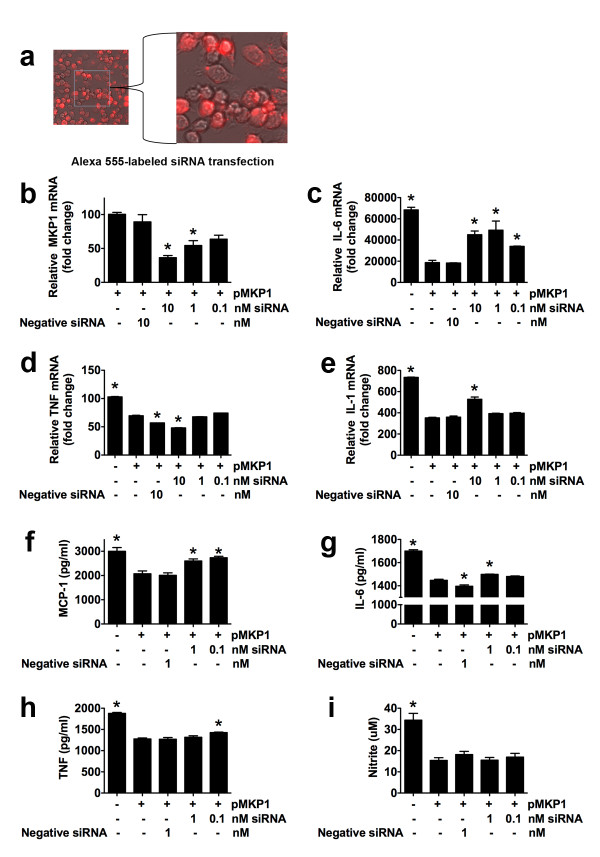
**Reversal effects of BV-2 cell MKP-1 induction in cytokine production by siRNA MKP-1 knockdown.** (**a**) Efficient siRNA BV-2 cell transfection using Alexa-555 red fluorescent-labeled siRNA. (**b**) MKP-1 mRNA level in BV-2 cells stably expressing MKP-1 after 24 h transfection with MKP-1 siRNA compared to negative control siRNA and vehicle treated cells. IL-6 (**c**), TNF-α (**d**), and IL-1β (**e**) mRNA quantification (qRT-PCR) in control (normal) and stably expressing MKP-1 BV-2 cells transfected 24 h with a negative siRNA, or a siRNA against MKP-1 and challenged with LPS (1 μg/ml) for 6 h (IL-6 and TNF-α) or 12 h (IL-1β). Since MKP-1 siRNA at 10 nM displayed an opposite effect than expected in TNF-α mRNA (potential non-specific effect), 1 nM was used for protein release experiments. Monocyte chemoattractant protein (MCP)-1 (**f**), IL-6 (**g**), TNF-α (**h**), and nitrite (**i**) release in control (normal) and stably expressing MKP-1 BV-2 cells transfected 24 h with a negative siRNA, or a siRNA against MKP-1 and challenged with LPS (1 μg/ml) for 6 h (MCP-1, IL-6, and TNF-α) or 12 h (nitrite). Results are expressed as mean ± s.e.m. of three experiments in duplicate. *P < 0.05 vs. BV-2 cells stably expressing MKP-1 (pMKP1 +).

We observed an increase in MKP-1 mRNA and protein in MKP-1 stably expressing cells (Figures 2a, 2b). These results also confirm the specificity of our antibody used for Western blot and immunofluorescence, since we observed that this MKP-1 antibody detected higher levels of MKP-1 protein in stably expressing MKP-1 BV-2 cells compared to normal BV-2 cells (Figure 2b). Overexpressing MKP-1 BV-2 cells challenged with lipopolysaccharide (LPS, 1 μg/ml) expressed lower levels of p-p38 (77 ± 4% and 77 ± 2% of control at 1 and 2 hr respectively, two replicates, Figure 2b) and p-JNK (80 ± 3% and 82 ± 3% of control at 1 and 2 hr respectively, two replicates, Figure 2b). Likewise, cells overexpressing MKP-1 that were challenged with LPS produced less IL-6, TNF-α, nitrite (nitric oxide metabolite) and monocyte chemoattractant protein (MCP)-1 as compared to control cells (normal, non-overexpressing MKP-1, LPS-treated BV-2 cells, Figures 2d–g). We also confirmed that ~90% of BV-2 cells were transfected with an Alexa-555 labeled siRNA (Figure 3a) and that a siRNA against MKP-1 reduced the MKP-1 expression in a concentration-dependent manner in BV-2 cells stably expressing MKP-1 (Figure 3b). This siRNA blocked the MKP-1-induced reduction of mRNA for IL-6 and IL-1β (Figures 3c, 3e) but not of mRNA for TNF-α (Figure 3d); it blocked MKP-1-induced reduction of MCP-1 and IL-6 (Figures 3f, 3g) but not of TNF-α or NO (Figures 3h, 3i). These results show the reliability of our MKP-1 cDNA since it demonstrates functionality. Therefore, we decided to use this MKP-1 cDNA in an *in vivo* setting.

### In vivo overexpression of MKP-1 in spinal cord of rats undergone L5 nerve transection

**Figure 4:**
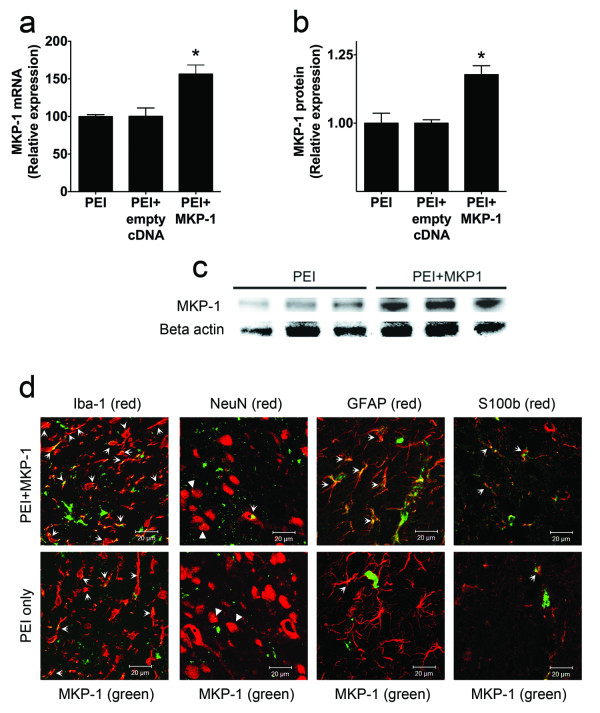
***In vivo*induction of spinal MKP-1.** Quantification of MKP-1 mRNA (*n* = 3) (**a**) and of MKP-1 protein (*n* = 3) (**b**), as well as a representative Western blot of MKP-1 expression (**c**), in L5–L6 spinal cord in rats that have undergone L5 nerve transection surgery and been treated i.t. with PEI only, PEI + empty cDNA, and PEI + MKP-1 cDNA four days after treatment and surgery. *P < 0.05 versus PEI only or PEI + empty vector. All data are expressed as mean ± s.e.m. (**d**) Representative images obtained by confocal microscopy and immunofluorescence depicting MKP-1 (in green) and the microglial cell marker Iba-1 (in red), the neuronal marker NeuN (in red), and the astrocytic cell markers GFAP (in red) and S100b (in red) in spinal cord of rats four days after L5 nerve transection and treated with PEI + MKP-1 cDNA or PEI only. Arrows indicate co-regionalization (in yellow/orange). Arrowheads show punctate co-regionalization in NeuN-expressing neurons.

We then sought to determine the feasibility of a non-viral vector nanoparticle system, polyethylenimine (PEI), for spinal cord tissue gene delivery [17]. PEI complexed with GFP cDNA administered intrathecally to naïve rats induced a cellular gene expression for two days (data not shown), in agreement with previous observations [17]. PEI complexed with MKP-1 cDNA (*N/P* = 15) injected i.t. in rats at the time of L5 nerve transection induced spinal MKP-1 mRNA and protein expression 4 days after surgery and treatment compared to the control groups (PEI only and PEI + empty vector pcDNA3.1) (Figures 4a–c). MKP-1 was expressed in Iba-1-expressing microglia, NeuN-expressing neurons, and GFAP- or S100b-expressing astrocytes (Figure 4d).

**Figure 5:**
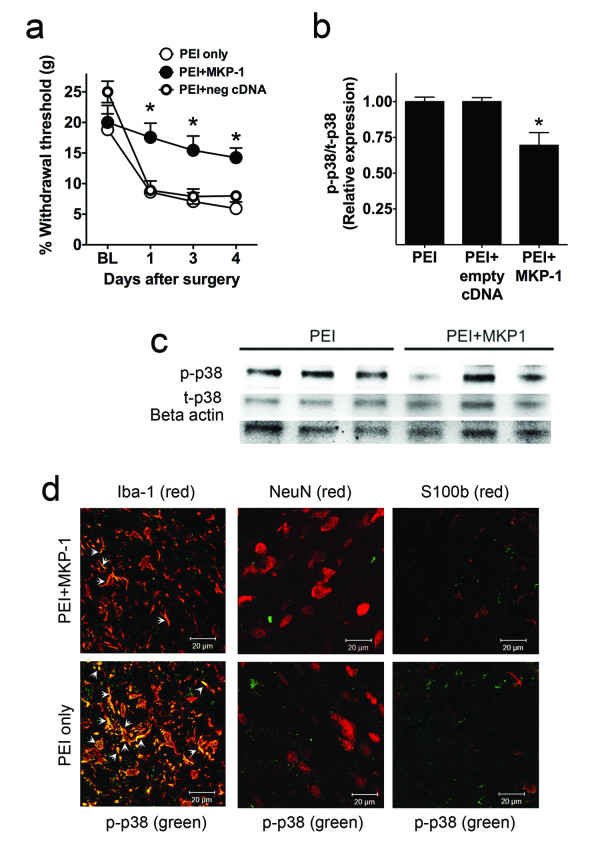
**Effects of*in vivo*induction of spinal MKP-1 in behavioral hypersensitivity and spinal p-p38 expression.** Withdrawal threshold to mechanical stimulation (**a**) in rats undergone L5 nerve transection surgery and treated i.t. with PEI only (*n* = 9), PEI + empty cDNA (*n* = 4), and PEI + MKP-1 cDNA (*n* = 9) four days after treatment and surgery. Quantification of p-p38 protein (**b**) and representative Western blot of p-p38 (**c**) expression from L5–L6 spinal cord of rats that have undergone L5 nerve transection surgery and been treated i.t. with PEI only (*n* = 3), PEI + empty cDNA (*n* = 3) and PEI + MKP-1 cDNA (*n* = 3) four days after treatment and surgery. *P < 0.05 versus PEI only or PEI + empty vector. All data are expressed as mean ± s.e.m. (**d**) Representative images obtained by confocal microscopy and immunofluorescence depicting p-p38 (in green) and the microglial marker Iba-1 (in red), the neuronal marker NeuN (in red), and the astrocytic marker S100b (in red) in spinal cord of rats four days after L5 nerve transection and treated with PEI + MKP-1 cDNA or PEI only. Arrows indicate co-regionalization (in yellow/orange).

The rats over-expressing MKP-1 (PEI + MKP-1 cDNA) did not develop mechanical allodynia for at least four days following peripheral nerve injury when compared to baseline and the PEI-only control rats (Figure 5a). In order to directly test that these results were due to MKP-1 expression, we also include a group with PEI + empty vector pcDNA3.1, which did not prevent the development of peripheral nerve-injury-induced hypersensitivity, in accordance with the lack of spinal cord overexpression of MKP-1 mRNA or protein of this group.

**Figure 6:**
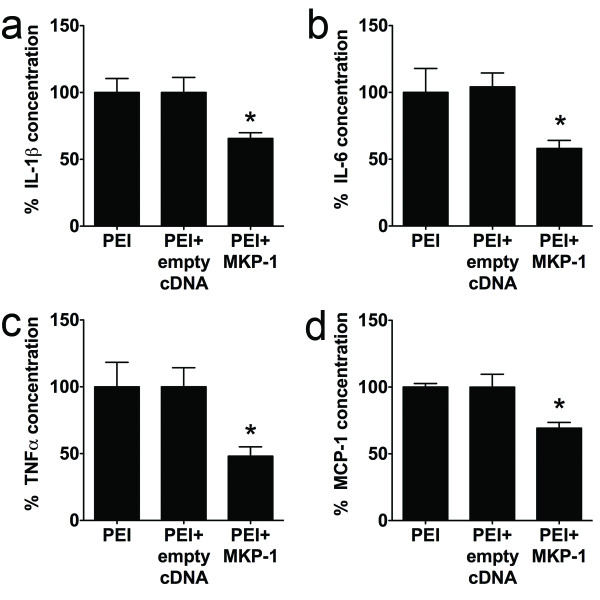
**Effects of*in vivo*induction of spinal MKP-1 in pro-inflammatory/algesic effectors.** Concentrations of IL-1β (**a**), IL-6 (**b**), TNF-α (**c**), and MCP-1 (**d**) from L5–L6 spinal cord of rats undergone L5 nerve transection surgery and treated i.t. with PEI only (*n* = 5), PEI + empty cDNA (*n* = 4), and PEI + MKP-1 cDNA (*n* = 9) four days after treatment and surgery. *P < 0.05 versus PEI only or PEI + empty vector. All data are expressed as mean ± s.e.m.

Spinal MKP-1-overexpressing rats also displayed lower levels of spinal p-p38 (Figures 5b5c), IL-1β, IL-6, TNF-α, and MCP-1 as compared to the PEI-only or PEI + empty vector groups (Figures 6 a–d). Phosphorylated p38 was preferentially expressed in Iba-1-expressing microglia, but not in S100b-expressing astrocytes or NeuN-expressing neurons (Figure 5d), in agreement with previous observations [13].

## Discussion

We have shown that spinal cord over-expression of MKP-1 reduces peripheral nerve-injury-induced spinal p-p38, presumably by dephosphorylation, which subsequently results in reduction of pro-inflammatory/pro-algesic effectors, preventing development of chronic allodynia.

Spinal cord MAPKs contribute to the perpetuation of chronic pain. Dual-specificity phosphatases (i.e., MKPs) dephosphorylate MAPKs, which reduces pro-inflammatory factor production and eventually induces resolution of inflammatory processes [18]. There are 16 identified dual-specificity MKPs [18]. MKP-1 is an immediate early gene product that preferentially dephosphorylates p-p38 over other MAPKs [18]. However, MKP-1 is also able to regulate p-ERK or p-JNK in some cases [18].

The acute and persistent spinal phosphorylation of p38 is instrumental in the development and maintenance of peripheral nerve-injury-induced allodynia [13]. In a rodent model of acute postoperative pain, skin-incision-induced p-p38 spontaneously returns to basal levels in parallel with resolution of allodynia [19]. The role of p-p38 in the initiation of acute postoperative pain has been demonstrated in this animal model of postoperative pain [19] and in humans [7]. Similarly, we have previously shown a functional association between persistent spinal p-p38 and persistent postoperative allodynia in a rat paw incision model [11]. Importantly, it has recently been shown that a p-p38 inhibitor is effective in reducing chronic pain in patients [8]. These data suggest that the transition from acute to chronic pain may be explained by an inability to restore basal MAPK phosphorylation of p-p38 in the spinal cord. Our data argue for this assumption. Spinal MKP-1 expression did not differ between peripheral nerve injury and naïve groups. However, the sham group displayed a higher level of spinal MKP-1 than the peripheral nerve injury group. These data suggest that skin incision results in an increase of spinal MKP-1, possibly to prevent p38 activation. In addition, peripheral nerve injury may limit MKP-1 expression after surgery, which allows p38 to be sustainably phosphorylated and contributes to peripheral nerve injury-induced allodynia. The therapeutic potential of p-p38 dephosphorylation by small molecules in chronic pain patients [8] also argues for the clinical relevance of our current findings and for the potential of MKP-1 as a target for the development of novel drugs to treat or prevent pain conditions.

Kinase phosphorylation requires adenosine triphosphate, while MKPs produce dephosphorylation by direct enzymatic action on the substrate, namely p-p38. This MKP mechanism of action is more efficient and may confer an enzymatic potency of 100–1000-fold over kinase activity [18]. This characteristic of MKPs makes them an attractive drug target, since modest induction of MKP expression may be a potent modulator of downstream pro-inflammatory molecules (e.g., MAPKs) [18]. In fact, the MKP-1 induction obtained with our approach was relatively modest (~25%), but this seems sufficient to significantly reduce p-p38 and cytokines in spinal cord and so produce overt and clear antinociceptive effects in a rat model of neuropathic pain. These findings are in accordance with the relatively higher potency of MKP-1 over the kinases that phosphorylate p38 [18]. This is relevant because neuropathic pain is one of the most difficult types of pain to treat in humans [2,3], and in general the hypersensitivity behaviors observed in rodent models of chronic pain [20] are more difficult to reverse than those displayed in rodent models of acute pain [21].

Phosphorylated p38 governs, at least in part, the production of microglial pro-inflammatory factors [9,10] believed to enhance activation of sensory neurons and sensitize the nociceptive system [22], contributing to rat-model behaviors suggestive of human pain [4,23]. Microglia, under inflammatory conditions, acquire an anti-inflammatory phenotype following MKP overexpression, manifested by a reduction of MAPK phosphorylation and downstream pro-inflammatory mediators such as cytokines or chemokines [10,14]. Therefore the effects of spinal MKP-1 induction in our current study may be a result of action in microglia. MKP-1 can produce similar anti-inflammatory effects in astrocytes and neurons; in fact, we did observe MKP-1 expression in these types of cells. However, downstream modulation of p-p38 by MKP-1 (p-p38 is the main substrate of MKP-1) was observed preferentially in microglial cells (Iba-1 expressing cells), in agreement with previous observations [13]. This finding argues for the potential relevance of microglial cell functions in the molecular and antinociceptive effects observed in this study.

We have previously show*n in vitro* that MKP-1 induction by a cannabinoid receptor type 2 agonist (JWH015) in primary microglial cells resulted in dephosphorylation of MAPKs and reduction of TNF-α production [14]. Others have shown similar findings with the endocannabinoid anandamide [24]. In that study, anandamide-induced MKP-1 reduced microglial NO release and was neuroprotective in a model of central inflammation. Similarly, others have shown that the induction of MKP-1 by dexamethasone induced p-38 dephosphorylation and a reduction of MCP-1 in rat primary microglia [10]. Spinal pro-inflammatory factors, such as TNF-α or MCP-1, contribute to generation of central sensitization and maintenance of chronic hypersensitivity [25-27]. Some of these pro-inflammatory factors, such as TNF-α, may cause direct nerve sensitization [28] and promote phosphorylation of spinal microglial p38, contributing to behavioral hypersensitivity in rodent models of neuropathic pain [9,29,30]. This reverberant pro-algesic loop between cytokines and MAPKs seems to be disrupted by the induction of MKP-1 in our study, which ultimately results in reduction of behavioral hypersensitivity induced by peripheral nerve injury. These earlier data, together with our current results, support the interpretation that MKP-1 is an important player in the mechanisms underlying chronic pain.

Since cytokines that are downstream p-p38 can also induce p38 phosphorylation [9,29,30], an alternative interpretation of our data may be that overexpressed MKP-1 directly reduces cytokine expression, and this may result in a reduction of p-p38. This possibility seems unlikely since MKP-1 is a enzyme with defined and identified substrates and actions: MAPKs and dephosphorylation respectively [18]. There is no literature description of phosphatases with direct effects on cytokines, which do not require phosphorylation to be active. Additionally, it is well established that cytokine production depends in part, and is downstream MAPK signaling pathways [9,10]. However, we recognize that the observed effects on cytokine production due to p38 dephosphorylation induced by MKP-1 overexpression may contribute to the overall reduction of p-p38 as a downstream mechanism.

*In vivo* spinal MKP-1 induction did not change spinal p-JNK expression (data not shown), but we did observe an increase in the phosphorylated form of extracellular signal-regulated protein kinase (ERK)-1/2 (data not shown). This is in agreement with the preference of MKP-1 to dephosphorylate p-p38 rather than p-ERK-1/2 [18]. Even though p-ERK signaling has been associated with allodynia, p-ERK is necessary for the MKP-1 structural stabilization, which prevents MKP-1’s early breakdown and enhances its activity [31,32]. Our data show the potentially greater relevance of spinal p-p38, compared to p-ERK, in the generation of peripheral nerve-injury-induced tactile allodynia.

In summary, our current results show that the spinal cord induction of MKP-1 is sufficient to prevent the development of peripheral nerve injury-induced allodynia for at least four days. Our study warrants further studies to determine whether these behavioral effects are only affecting the mechanisms governing the development of chronic allodynia or are also blocking the mechanisms of chronic hypersensitivity. Likewise, our study warrants further investigation on the potential role of other phosphatases in pain states, and the potential role of MKP-1 in other types of pain, such as acute pain.

## Conclusions

In conclusion, we have uncovered that the immunomodulatory molecule MKP-1, which is involved in the resolution of inflammatory processes in the periphery, is also able to resolve the spinal pro-inflammatory phenotype induced by peripheral nerve injury. We demonstrated that MKP-1 retains its substrate specificity, namely p-p38, in spinal cord (as also observed in other systems) and that dephosphorylation of p-p38 may explain the reduction of spinal pro-algesic factors (cytokines and chemokines) and mechanical hypersensitivity in a rat model of neuropathic pain. We identified MKP-1 as a potential new target for drug development. The clinical utility of MKP-1 inducers is supported by the greater potency of MKPs over kinase activity, which will theoretically possess analgesic efficacy with modest molecular effects as compared to current p-p38 inhibitors. The clinical potential of p-p38 modulators has already been shown; our findings may provide the foundation for the development of novel drugs with more specific MKP-1 induction than cannabinoids or glucocorticoids.

## Methods

### Cloning of the rat MKP1 gene

Spinal cord tissue from Sprague Dawley rats (Harlan, Indianapolis, IN, 250 g) was harvested and stored at –80°C. Frozen tissues were homogenized and total RNA was extracted using RNA easy columns (Qiagen, Basel, Switzerland), following the manufacturer’s protocol. Total RNA was stored at –20°C until further use. Single strand cDNA was synthesized from 1 μg of rat spinal cord total RNA using the Quantitect reverse transcription kit (Qiagen, Basel, Switzerland), following the manufacturer’s protocol. For MKP-1 polymerase chain reaction (PCR), forward (5’CGACAGACTATTTCTGGAGAGCTGC3’) and reverse (5’CGGTGGCACGTGAAACTCCACA3’) primers were designed to be homologous to the rat dual-specificity phosphatase 1 gene (Genbank accession number NM_053769). These primers were used to generate 1.198 kb PCR fragment from spinal cord cDNA samples. PCR was performed as follows: 95°C for 5 min followed by 30 cycles at 95°C for 15 sec, 61°C for 1 min, 72°C for 1 min 15 sec, and 72°C for 10 min. The PCR product was gel-purified and cloned into pcDNA3.1 expression vector (Invitrogen, San Diego, CA). The MKP-1 gene was sequenced on both strands using the Big Dye terminator v3.1 cycle sequencing kit (Applied Biosystems, Foster city, CA) and confirmed the expected rat MKP-1.

### Cell culture, transfection, and lipopolysaccharide (LPS) treatment

The immortalized mouse microglial cell line BV-2 (a generous gift of Dr. Weihua Zhao, Methodist Hospital, Houston, TX) was cultured in DMEM (Mediatech, Manassas, VA) supplemented with 10% charcoal-stripped FBS (Hyclone), 1.1% GlutaMax (Invitrogen, San Diego, CA), and 1% Penicillin-Streptomycin (100 U/ml penicillin, 100 μg/ml streptomycin, Mediatech) and maintained in 5% CO_2_ at 37°C. BV-2 cell viability was assessed by trypan blue staining (Sigma, St. Louis, MO) and hemocytometer. A cell line stably expressing MKP-1 was created by transfection of rat MKP-1 (5 μg) cDNA into 1x10^6^ BV-2 by electroporation (Lonza Biologics, Basel, Switzerland) following manufacturer’s instructions. After 48 h, cells were selected in growth medium containing neomycin (G418 sulfate, 800 μg/ml) for about 2 weeks. Stable transformants were then split at different dilutions in plates containing growth medium and neomycin selection drug. Cells were challenged with 1 μg/mL of LPS (0111:B4 serotype, Sigma, St. Louis, MO) in complete media for 1, 2, 6, and 12 h. Following LPS treatment, supernatants were removed and stored at –80^o^C until nitric oxide (NO) and cytokine measurement. Cells were used for quantitative RT-PCR and Western blot analyses.

The stealth siRNA oligonucleotides were synthesized by Invitrogen (Invitrogen, San Diego, CA) with sequence complementary to rat MKP-1 5’UCGUGAAGCAGAGGCGGAGUAUUAU3’ and 5’AUAAUACUCCGCCUCUGCUUCACGA3’. The stealth siRNA negative control duplex was used as control oligonucleotide (5’UUCCUCUCCACGCGCAGUACAUUUA3’ and 5’UAAAUGUACUGCGCGUGGAGAGGAA3’). Transfection efficiency (~85%) was monitored using BLOCK-iT Alexa Fluor red fluorescent oligo (Invitrogen, San Diego, CA). Stealth MKP-1 siRNAs (0.1, 1, and 10 nM) were transfected for 24 hr in BV-2 stably expressing MKP-1 using RNAi max transfection reagent (Invitrogen, San Diego, CA), following manufacturer’s instructions.

### Quantitative real time (RT)-PCR

For mRNA quantification, total RNA isolated from BV-2 was reverse transcribed into single-strand cDNA as described above. Quantitative RT-PCR was performed using a 96-well plate with the Applied Biosystems 7500 Real-Time PCR system (Applied Biosystems, Foster city, CA) according to the following conditions: 1 cycle of 50°C for 2 min, 1 cycle of 95°C for 10 min, then 40 cycles at 95°C for 15 sec, 60°C for 1 min. All samples were run in duplicate using Taqman gene expression assays (Applied Biosystems, Foster City, CA) with rat MKP-1 (Rn00678341_g1) predesigned and preformulated primer and probe. Expression of MKP-1 was normalized to a predesigned and preformulated rat (Rn01775763_g1) or mouse (Mm99999915_g1) control gene GAPDH (Applied Biosystems, Foster City, CA). The difference in MKP-1 mRNA expression between treatments was analyzed using the comparative C_T_ method. The threshold cycle (C_T_) is defined as the cycle at which the amount of amplified PCR product from the target cDNA reaches a fixed threshold. In each treatment, ΔC_T_ = MKP1C_T_ – C_T_ for the endogenous reference, GADPH. ΔΔC_T_ = ΔC_T__treatment_ – ΔC_T__control_. The N-fold of differential expression of MKP-1 transcript in the treated group compared to that in the control group is expressed as 2^- ∆∆CT^. This number is converted to percent of control, where control is set at 100. Total RNA for each sample was also included during each run as a negative control. Predesigned and preformulated primers and probes for rat and mouse cytokines interleukin (IL)-6, tumor necrosis factor (TNF)-α, and IL-1β mRNAs—i.e., for rat, Rn 99999011_m1, Rn 99999017_m1, and Rn 00580432_m1 respectively, and for mouse, Mm 99999064_m1, Mm 00443258_m1 and Mm 01336189_m1 respectively—were used for quantitative RT-PCR as described above.

### Western blot analyses

BV-2 cell protein or hemi-sectioned spinal cord (ipsilateral to injury) was collected in 100 μL of 1x Laemmli buffer (Bio-Rad, Hercules, CA) containing either 5% 2-β Mercaptoethanol (Sigma, St. Louis, MO). Protein (40–50 μg) or standard protein markers were subjected to SDS polyacrylamide gel electrophoresis (10% or 18% gels, Bio-Rad, Hercules, CA) and transferred to polyvinylidene difluoride (PVDF, Bio-Rad, Hercules, CA) membranes. Membranes were blocked with 5% BSA in TBS-Tween 20 (0.05%, Sigma, St. Louis, MO), then incubated overnight at 4°C with mouse anti rat MKP-1 monoclonal antibody (1:500, Santa Cruz Biotechnology, Santa Cruz, CA), mouse anti-phospho-ERK 44/42 (Phospho-MAP Kinase 1:500, Cell Signaling, Danvers, MA), mouse anti-phospho-p38 (1:500, Cell Signaling, Danvers, MA), or mouse anti-phospho-JNK (1:500, Cell Signaling, Danvers, MA). Blots were incubated with goat HRP-conjugated secondary antibody (1:3000, Pierce, Rockford, IL), treated with SuperSignal West Femto Maximum Sensitivity Substrate (Thermo Fisher Scientific, Rockford, IL), and imaged with Syngene G-box (Synoptics, Frederick, MD). Membranes were stripped and re-probed with rabbit anti-ERK 44/42 (Total MAP Kinase 1:500, Cell Signaling, Danvers, MA), rabbit anti-p38 (total MAP kinase 1:500, Cell Signaling, Danvers, MA), rabbit anti-JNK (1:500, Cell Signaling, Danvers, MA), or mouse anti-*β*-actin antibody (1:3000, Abcam, Cambridge, MA). Band density was quantified in relation to loading controls using Syngene Tools software (Synoptics, Frederick, MD). MAPKs were evaluated at 1 and 2 hr following LPS stimulation in BV-2 cells, since these early activated molecules are upstream cytokines, chemokines and nitric oxide production (which were measured at later time points, 6 and 12 hr after LPS stimulation in BV-2 cells)**.**

### Griess assay (Nitric oxide production) and ELISA assays

Supernatants collected from LPS-treated cells or total proteins (20 μg) extracted from L5 spinal cord were assayed for (1) nitric oxide (NO), measured as nitrite, using the Griess Assay (Promega, Madison, WI), (2) MCP-1 using the BD OptEIA™ ELISA kits for mouse and rat MCP-1 (BD Biosciences, San Diego, CA), and (3) IL-1β, IL-6, and TNF-α using the respective R&D Systems DuoSet ELISA (R&D Systems, Minneapolis, MN) kits following the manufacturer’s protocol.

### Animals, vector delivery, surgeries, and behavior testing to assess allodynia

Sprague-Dawley rats weighing 250–300 g (Harlan) at the start of the study were housed individually and maintained in 12:12 h light-dark cycle with *ad libitum* access to food and water. All procedures were approved by the Institutional Animal Care and Use Committee at Dartmouth College (Dartmouth Medical School, Hanover, New Hampshire) and in accordance with the Guidelines for Animal Experimentation of the International Association for the study of Pain. Plasmid DNA expressing MKP-1 (pcDNA-MKP-1) or empty plasmid DNA (pcDNA3.1) used in this study was purified using an endotoxin-free Qiagen plasmid purification kit and resuspended in sterile water. After determining the concentration of pcDNA-MKP-1 using 260 nm adsorption, aliquots of 8 μg were stored at –20°C. The purity of pcDNA-MKP-1 was determined by 260:280 nm adsorption and ranged between 1.9 to 2.0.

We used the *in vivo* non-viral nanoparticle preparation polyethylenimine (PEI), in vivo-JetPEI (PEI, Polyplus Transfection, New York, NY) for our *in vivo* gene transfection and induction. Endotoxin-free PEI is a cationic polymer that protects nucleic acids from degradation when administered in vivo [33,34]. The positively charged PEI-nucleic acid complex interacts with the cellular membrane anionic proteoglycans, facilitating entry of the molecule into cells by endocytosis [35]. Genes complexed with PEI have shown efficient induction into the brain and spinal cord parenchyma (glial and neuronal) of mice and rats when administered intrathecally or intracerebrally [36-39]. This CNS tissue penetration is not achieved with microparticles limited to the meningeal tissue following intrathecal administration [40]. CNS administration of PEI nanoparticles with DNA plasmids has shown minimal toxicity, no necrosis [36], no inflammatory response [41], and no overt side effects in CNS animal transfections [42]. Additionally, PEI transfection does not alter the electrophysiological properties of neurons [43], which makes this nanoparticle transfection agent a suitable tool for pain research.

On the day of intrathecal (i.t.) injection, aliquot of pcDNA-MKP-1 or empty pcDNA3.1 was thawed on ice and complexed with in vivo-JetPEI as described by the manufacturer: 8 μg of pcDNA plus in vivo-JetPEI in 5% glucose in a final ratio of nitrogen residues of in vivo-JetPEI per nucleic acid phosphate of 15 (*N/P* = 15) and a total volume of 20 ul. The preparation was incubated at room temperature for 15 min before the injections. The ready complex PEI + pcDNA-MKP1 (PEI + MKP-1, *n* = 9 animals), PEI + pcDNA alone (empty cDNA, *n* = 4 animals), or PEI alone (*n* = 9 animals) was administered by lumbar-puncture i.t. injection using a Hamilton syringe and a 25-gauge 5/8 hypodermic needle, as we have described before [20], under brief inhalational anesthesia (2–3% isoflurane in oxygen). The needle was inserted intrathecally, on the midline between the fourth and fifth lumbar vertebrae. Injection site was confirmed by stimulation of nerves in the cauda equina when the needle penetrated the dura and manifested with a brief and obvious movement of the tail and/or the hind paws. The animals regained consciousness 2–3 min after discontinuation of anesthesia.

Animals underwent L5 nerve transection (L5NT) procedure as previously described [20]. Briefly, rats were anesthetized with 2% isoflurane in oxygen and a small incision was made to the skin overlying L5–S1, followed by retraction of the paravertebral musculature. The L6 transverse process was then removed and the L5 spinal nerve identified, lifted slightly, and transected. The wound was sutured. Sham surgeries were performed similarly, except for nerve manipulation or transection. Intrathecal injection of PEI + cDNAs or PEI only was performed immediately after surgery.

Behavioral testing was performed to assess the development of mechanical allodynia before injection or surgery and on postoperative days 1–4 [20]. The withdrawal threshold to mechanical stimuli was assessed ipsilaterally to surgery using calibrated von Frey filaments (Stoelting, Wood Dale, IL) and an up-down statistical method [44]. The investigator was blinded to treatment in all behavioral tests.

### Immunohistochemistry

Rats were deeply anesthetized with 2–3% isoflurane in oxygen and transcardially perfused with 10 mM phosphate buffer saline (200 mL) followed by 4% formaldehyde (400 mL) at room temperature. The L5 portion of the spinal cord was removed and placed in 30% sucrose for 48–72 h at 4°C. The tissue was then frozen at –80°C in optimal cutting temperature compound (Sakura Finetek, Torrance, CA). Immunohistochemistry was performed on transverse 20 μm L5 spinal cord free-floating sections. The sections were blocked for 1 h in 5% normal goat serum (NGS, Vector Labs, Burlingame, CA) and 0.01 Triton X-100 (Sigma) in PBS. Then the sections were incubated overnight in mouse anti rat monoclonal antibody for MKP-1 (1:100, Cell Signaling, Danvers, MA). The next day, a TSA Signal Amplification Kit (PerkinElmer LifeSciences Inc, Boston, MA) was used, following the manufacturer’s instructions, to enhance the visualization of MKP-1 expression. Sections were washed 2 times for 5 min in PBS then incubated in a biotinylated Goat anti-rabbit secondary antibody for 1 h at 4°C. Sections were then washed, incubated in SA-HRP (1:100) for 1 h at 4°C, washed again and incubated in the TSA fluorophore (1:250) for 10 min at 4°C. Sections were then washed again and incubated overnight in rabbit anti rat antibody for ionized calcium binding adaptor molecule 1 (Iba-1, 1:1000, microglia marker, Wako, Richmond, VA), rabbit anti glial fibrillary acidic protein (GFAP, 1:10,000, astrocyte marker, Dako, Carpinteria, CA), rabbit anti S100 calcium binding protein B (S100B, 1:15,000, astrocyte marker, Fitzgerald, Concord, MA), or rabbit NeuN/Fox3 (NeuN, 1:10,000, neuron nuclear marker, Biosensis, Australia). The next day sections were washed three times, then incubated for 1 h in goat anti-mouse Alexa 488 IgG_3_ and goat anti-rabbit Alexa 555 IgG secondary antibodies (1:250, Invitrogen). Tissue sections were washed, mounted on glass slides, dehydrated, treated with Vectashield (Vector Labs, Burlingame, CA), and sealed with a coverslip and nail polish. The specificity of each antibody was tested by omitting the primary antibody on 1–3 additional sections. All MAPK and cell marker antibodies are widely used for their specificity. We confirmed the specificity of our MKP-1 antibody by using Western blots in MKP-1 overexpressing BV-2 cells and normal BV-2 cells (see Results section). The following groups were included to test MKP-1 antibody signal amplification using the TSA kit: (1) only the anti-MKP-1 primary antibody (1:100) and the Alexa 555 Goat anti-rabbit secondary antibody, (2) only the anti-MKP-1 primary antibody and the TSA kit, and (3) the TSA kit, the cell marker primary, and the Alexa 555 Goat anti-rabbit secondary antibody, but excluding the anti-MKP-1 primary antibody. All controls confirmed the specificity of the complete co-stain. Sections for single antibody immunofluorescence were imaged with a Q-Fired cooled CCD camera attached to an Olympus microscope. Confocal microscopy of dual antibody immunofluorescence was performed with a Zeiss LSM 510 Meta confocal microscope (Englert Cell Analysis Laboratory of Dartmouth Medical School), and images were prepared with the Zeiss LSM software (Thornwood, NY) and Adobe Photoshop software (San Jose, CA). All images were taken from laminae I–II of dorsal horn spinal cord ipsilateral to nerve injury.

### Statistical analysis

All *in vitro* experiments were completed at least three time. Data are expressed as mean ± s.e.m. Statistical analyses were completed using GraphPad Prism 5 (GraphPad Software, Inc., San Diego, CA). One- or two-way ANOVA and Bonferroni post test were used when appropriate. Unpaired t-test was use for MKP-1 mRNA expression and cytokine release. Significance was determined at a level of *P* < 0.05 or with exact *p* value where available.

## Abbreviations

MKP, Mitogen-activa ted protein kinase-phosphatase; PCR, Polymerase chain reaction; cDNA, Complementary DNA; PEI, Polyethylenimine; LPS, Lipopolysaccharide; ERK, Extracellular signal-regulated kinase; p-ERK, Phospho-extracellular signal-regulated kinase; t-ERK, Total-extracellular signal-regulated kinase; JNK, c-Jun N terminal kinase; p-JNK, Phospho-c-Jun N terminal kinase; t-JNK, Total-c-Jun N terminal kinase; p-p38, Phospho-p38; Il, Interleukin; TNF, Tumor necrosis factor; MCP, Monocyte chemoattractant protein; NO, Nitrite oxide; L5NT, L5 nerve transection; siRNA, Small interfering RNA; GAPDH, Glyceraldehyde 3-phosphate dehydrogenase.

## Competing interests

The authors declare that they have no competing interests.

## Authors’ contributions

EAR-S participated in the immunohistological studies, carried out behavioral studies, conceived the study, participated in statistical analyses and participated in its design and coordination and drafted the manuscript. CN participated in the cell culture preparations, participated in the molecular studies, developed the genetic tools, participated in the experimental design and statistical analyses and helped to draft the manuscript. RPL participated in the cell culture preparations, immunohistochemistry and molecular studies, and helped to draft the manuscript. JAD participated in the study design and coordination and edited the manuscript. All authors read and approved the final manuscript.
